# Outcomes of intensive care patients with acute small-vessel vasculitis: a 23-year single-centre experience

**DOI:** 10.1093/rheumatology/keag531

**Published:** 2026-09-22

**Authors:** Arden Dierker Viik, Yiwang Xu, Dominic McGovern, Seerapani Gopaluni, Michael Chen-Xu, James Varley, Lisa Willcocks, Rona Smith, Iftach Sagy, David Jayne, Rachel Jones

**Affiliations:** School of Clinical Medicine, University of Cambridge, Cambridge, UK; School of Clinical Medicine, University of Cambridge, Cambridge, UK; Department of Medicine, University of Cambridge, Cambridge, UK; Vasculitis and Lupus Clinic, Addenbrooke’s Hospital, Cambridge, UK; Department of Medicine, University of Cambridge, Cambridge, UK; Department of Medicine, University of Cambridge, Cambridge, UK; Vasculitis and Lupus Clinic, Addenbrooke’s Hospital, Cambridge, UK; Intensive Care Unit, Addenbrooke’s Hospital, Cambridge, UK; Vasculitis and Lupus Clinic, Addenbrooke’s Hospital, Cambridge, UK; Department of Medicine, University of Cambridge, Cambridge, UK; Vasculitis and Lupus Clinic, Addenbrooke’s Hospital, Cambridge, UK; Department of Medicine, University of Cambridge, Cambridge, UK; Vasculitis and Lupus Clinic, Addenbrooke’s Hospital, Cambridge, UK; Department of Medicine, University of Cambridge, Cambridge, UK; Vasculitis and Lupus Clinic, Addenbrooke’s Hospital, Cambridge, UK; Department of Medicine, University of Cambridge, Cambridge, UK; Vasculitis and Lupus Clinic, Addenbrooke’s Hospital, Cambridge, UK

**Keywords:** small-vessel vasculitis, ANCA-associated vasculitis, anti-glomerular basement membrane disease, intensive care, rituximab, cyclophosphamide, plasma exchange, mortality, end-stage kidney disease

## Abstract

**Objectives:**

Patients with fulminant small-vessel vasculitis (SVV) may require intensive care unit (ICU) admission, where mortality is high. We describe outcomes and prognostic factors in ICU patients with ANCA-associated vasculitis (AAV) and anti-glomerular basement membrane (anti-GBM) disease over a 23-year period.

**Methods:**

Patients admitted with acute manifestations of new or flaring AAV or anti-GBM disease were identified from our hospital ICU database (1999–2022). Patients treated solely in high-dependency areas were excluded. Primary outcomes were mortality at 30 and 90 days and development of end-stage kidney disease (ESKD). A matched case–control analysis (1:4) adjusted for age, gender, APACHE II score and admission year was performed for a patient subgroup.

**Results:**

Eighty-five cases (82% AAV, 15% anti-GBM) were identified, 80% newly diagnosed. Median APACHE II score was 19. Ventilatory support and continuous venovenous haemodiafiltration were required in 92% and 66% of cases, respectively. Thirty-day and 90-day mortality rates were 15% and 26%, respectively. Among 90-day survivors, 17 (28%) developed new ESKD. Vasculitis treatments included cyclophosphamide (59%), rituximab (53% of AAV patients) and plasma exchange (71%). These treatments were associated with 90-day and long-term survival. Treatment for vasculitis post-2008 was associated with 90-day and long-term survival, which was not mirrored in ICU controls. This coincided with improvements to the vasculitis service and therapeutic strategies.

**Conclusion:**

This single-centre study reports ICU outcomes for patients with fulminant SVV over 23 years. Thirty-day and 90-day ICU mortality were comparable to other reported values. Improvement in mortality was associated with changes in vasculitis treatment protocols.

*Rheumatology* key messages

- ICU patients with acute small-vessel vasculitis had 15% 30-day and 26% 90-day mortality.
- Among ICU survivors with small-vessel vasculitis, 28% developed new end-stage kidney disease.
- Survival in ICU patients with small-vessel vasculitis improved after 2008 alongside service changes.

## Introduction

Anti-neutrophil cytoplasmic antibody (ANCA)-associated vasculitis (AAV), and anti-glomerular basement membrane (anti-GBM) disease are rare small vessel vasculitides (SVV) associated with life threatening manifestations [1]. AAV includes three clinically distinct syndromes; granulomatosis with polyangiitis (GPA), microscopic polyangiitis (MPA) and eosinophilic granulomatosis with polyangiitis (EGPA). AAV and anti-GBM, together referred to as SVV, have a propensity for pulmonary and renal organ-threatening manifestations. Acute presentations of SVV may result in rapid clinical deterioration and patients may require intensive care unit (ICU) treatments including mechanical ventilation, continuous venovenous haemodiafiltration (CVVHDF) and cardiovascular support.

Both ICU management and vasculitis-specific treatments have evolved over the past two decades, with technological advances and availability of new therapeutic agents. Steroids and cyclophosphamide are used for induction in SVV [2], with rituximab additionally introduced for the treatment of AAV during this time [3]. Plasma exchange removes pathogenic autoantibodies and can serve as an adjunct to treatment in SVV. However, plasma exchange results in loss of humoral immunity and increases infection risk in an already vulnerable patient population. The PEXIVAS study [4] found that the use of plasma exchange did not improve mortality in AAV patients, but it has been found to be for beneficial for anti-GBM disease [5, 6].

Only a small number of studies have reported outcomes of patients admitted to ICU with acute manifestations of SVV [7–16], with mortality rates of up to 61% reported. The small size of these studies prohibited the correlation between many variables and mortality, but the significance of severity of illness at ICU presentation was clear, with Acute Physiology and Chronic Health Evaluation (APACHE) [17] score or Birmingham Vasculitis Activity Score (BVAS) [18] often associated with higher mortality.

In this 23-year, single-centre, retrospective study, the characteristics, prognostic factors and outcomes of patients with acute SVV on ICU at Addenbrooke’s Hospital in Cambridge (a tertiary referral centre in the UK) are explored.

## Materials and methods

### Assembly of cohort

An observational cohort of Vasculitis Intensive Care Unit (VICU) patients without internal controls was collected for primary analyses. Patients were eligible for inclusion if they were 16 years or older and were admitted to the ICU or the Neurosciences Critical Care Unit (NCCU) at Cambridge University Hospitals for the management of SVV. Patients with vasculitis in remission who were admitted to ICU for unrelated reasons were not included. For patients with multiple ICU admissions, only the first encounter was considered. Eligible patients had either a new diagnosis of SVV, defined as a diagnosis made during hospitalization or within 1 month of starting remission-induction treatment; progressive disease, defined as clinical worsening within 2–6 months of starting remission-induction therapy; refractory disease, defined as receiving vasculitis treatment for more than 6 months without achieving remission; or relapsed disease, defined as more than 6 months since the initial diagnosis with at least one previous period of remission. Patients requiring only high-dependency care or single-organ support, such as haemodialysis or non-invasive ventilation, were not included.

Eligible patients were primarily identified through ICU and NCCU admission records between January 2000 and December 2022. Additional sources were reviewed to ensure complete case capture, including plasma-exchange records, intravenous immunoglobulin (IVIg) logs, clinical-trial databases and ICU telephone-referral records, to minimize the risk of missed cases due to miscoding.

For a separate analysis, a control cohort of ICU patients without vasculitis (non-VICU) was assembled from ICU admissions between 1999 and 2017 and matched to cases in a 1:4 ratio by age, gender, APACHE II score and year of ICU admission.

### Study outcomes

The primary aim of the study was to determine all-cause mortality rates at 30 and 90 days from the time of ICU admission and to establish the incidence of end-stage kidney disease (ESKD). Patients were considered to have developed ESKD if they survived to 90 days, had not required renal-replacement therapy before the hospitalization that led to ICU admission, and remained dialysis-dependent 90 days after ICU admission. Long-term mortality and renal outcomes were collected until death or the end of follow-up, with a maximum follow-up duration of 65 months, for use in time-to-event analyses.

Secondary aims were to characterize the local SVV cohort, identify predictors of mortality and ESKD, and review vasculitis-treatment practices in the acute setting. Clinical manifestations of vasculitis were collected to support these analyses. Probable renal vasculitis was defined as haemoproteinuria on urinalysis or evidence of impaired renal clearance in the context of active systemic SVV, with biopsy confirmation where available. Pulmonary haemorrhage was recorded if haemoptysis was present, if bronchoscopy demonstrated pulmonary bleeding or if imaging showed features consistent with alveolar haemorrhage.

#### Statistical analysis

Continuous variables were expressed as medians (interquartile range) or mean (95% confidence interval). Categorical variables were presented as percentages and frequencies. Statistical tests used to assess risk of 30-day mortality, 90-day mortality and renal replacement therapy (RRT) dependency at 90 days were chosen to reflect the data type and were conducted on both the VICU cohort and a sub-cohort of AAV patients. Between-group comparisons for continuous variables were performed using the Mann–Whitney *U*-test (non-normal distribution) or Student’s independent *t*-test (normal distribution), and on discrete variables using the chi-squared or Fisher’s exact test. Binomial logistic regression analyses were performed to identify predictors of 30-day mortality, 90-day mortality and ESKD. Multivariable models for these were constructed by including covariates that were statistically significant in the univariable analysis and/or clinically relevant to the outcomes in question. Survival distributions were assessed using the log-rank test. Cox proportional hazards regression analysis was performed for long-term mortality alone. This analysis included variables that were clinically meaningful and was refined further using Schoenfeld residuals. For the ICU control analysis, cases were matched using the case-control matching function in SPSS version 25 (IBM Corp., Armonk, NY, USA), and *P*-values <0.05 were considered statistically significant. Statistical analyses were carried out with SPSS version 25 and R version 4.3.1.

#### Ethics

This study was formally registered and approved as a service evaluation at Cambridge University Hospitals NHS Foundation Trust. As a service evaluation using retrospectively collected data from electronic health records, NHS Research Ethics Committee approval and individual patient consent were not required.

## Results

Eighty-five patients (57 men and 28 women) with a median age of 63 years (IQR 48–71 years) were admitted to ICU for the management of vasculitis from January 2000 to December 2022. The VICU patient characteristics and ICU admission indications are summarized in Table 1.

**Table 1:** VICU population characteristics and ICU management details including specific treatment.

Characteristic	Value (*n* = 85)
Patient characteristics	
Age on ICU admission, median (IQR)	63 (48–71)
Male gender, *n* (%)	57 (67)
Co-morbidities, *n* (%)	
Previous ESKD^a^	5 (6)
Hypertension^b^	31 (36)
Other cardiovascular diseases^b^	11 (13)
Chronic lung disease^b^	20 (24)
Diabetes mellitus^c^	9 (11)
ICU admission	
Previous admission to Cambridge ICU/NCCU^d^, *n* (%)	3 (4)
ICU stay length, median (IQR), days	8 (3–19)
Follow-up duration, median (IQR), months	17 (1–65)
APACHE II score on admission^e^, median (IQR)	19 (15–23)
ICU indications, *n* (%)	
Respiratory failure	73 (86)
Renal failure	55 (65)
Of which pulmonary-renal syndrome	49 (89)
Cardiovascular instability	19 (22)
Neurological instability	8 (9)
Gastrointestinal manifestations	9 (11)
Sepsis	2 (2)
Other	4 (5)
ICU interventions, *n* (%)	
Ventilatory support	78 (92)
Of which invasive ventilation^a^	47 (60)
CVVHDF^a^	56 (66)
Inotropes^f^	33 (39)
ECMO^g^	5 (6)
Vasculitis characteristics	
Diagnosis^a^, *n* (%)	
AAV	70 (82)
GPA	45 (64)
MPA	23 (33)
EGPA	2 (3)
Anti-GBM	13 (15)
Others	1 (1)
Disease onset, *n* (%)	
New	68 (80)
Progressive	3 (4)
Refractory	1 (1)
Relapsed	13 (15)
Renal manifestations, *n* (%)	
Renal biopsy performed	25 (29)
Of which confirmed renal vasculitis	22 (88)
Probable renal vasculitis^b^	63 (74)
Of which needing CVVHDF^c^	52 (83)
Pulmonary manifestations, *n* (%)	
Haemoptysis^c^	39 (46)
Imaging evidence of alveolar haemorrhage	56 (66)
Bronchoscopy performed^a^	38 (45)
Of which confirmed haemorrhage	30 (79)
Combined evidence of pulmonary haemorrhage^a^	62 (73)
Of which needing invasive ventilation^d^	37 (60)
Vasculitis treatment, *n* (%)	
Induction therapy	
Cyclophosphamide	50 (59)
Rituximab^e^	37 (44)
Also receiving cyclophosphamide	19 (51)
Other agents^f^	12 (14)
Adjunctive therapy	
Plasma exchange	60 (71)
Also receiving rituximab	32 (53)
Intravenous immunoglobulins	6 (7)
Key categories of adverse events in ICU, *n* (%)	
Sepsis/need for intravenous antibiotics	15 (18)
Progressive bleeding	9 (11)

a One missing.

b Six missing.

c Five missing.

d Eight missing.

e Ten missing.

f Two missing.

g The first ECMO case was in 2010.

h One missing.

i Probable renal vasculitis includes biopsy proven cases (*n* = 22), and cases diagnosed on the basis of abnormal renal function and or abnormal urinary sediment (*n* = 41).

j A total of 56 VICU patients received RRT, 93% (52 of 56) for probable renal vasculitis and 7% (4 of 56) for other causes of renal failure.

k A total of 47 VICU patients received invasive ventilatory support, 79% (37 of 47) for pulmonary haemorrhage and 21% (10 of 47) for other causes of respiratory failure.

l Rituximab was only given to patients with ANCA-associated vasculitis.

m Including mycophenolate, azathioprine, adalimumab, alemtuzumab and erlizumab. Abbreviations: AAV: ANCA-associated vasculitis; anti-GBM: anti–glomerular basement membrane disease; APACHE II: Acute Physiology and Chronic Health Evaluation II; CVVHDF: continuous venovenous haemodiafiltration; ECMO: extracorporeal membrane oxygenation; EGPA: eosinophilic granulomatosis with polyangiitis; ESKD: end-stage kidney disease; GPA: granulomatosis with polyangiitis; ICU: intensive care unit; IQR, interquartile range; MPA: microscopic polyangiitis; NCCU: neurosciences critical care unit; RRT: renal replacement therapy; VICU: vasculitis-related intensive care unit.

Respiratory failure was the most common primary reason for VICU admission, with 55% of the cohort requiring invasive ventilation. Renal failure was the second most common reason for VICU admission. Of patients with renal failure, 89% had concurrent pulmonary failure, reflecting the need for multi-organ support in this cohort.

Table 1 describes the manifestations of vasculitis in this cohort as well as the treatment provided. The most common diagnosis was GPA, followed by MPA, anti-GBM patients and finally EGPA. Most patients (80%) presented to ICU with a new diagnosis of SVV.

Twenty-five patients had a renal biopsy, which confirmed 22 cases of renal vasculitis. Renal vasculitis was diagnosed in 63 patients (74%) reflecting diagnosis from methods other than biopsy: abnormal renal clearance or dipstick haemoproteinuria in the context of active systemic SVV, judged by a specialist vasculitis service. Pulmonary haemorrhage was a common finding in this patient group, and 62 patients (73%) had evidence of pulmonary haemorrhage.

To treat vasculitis, all patients received pulsed intravenous glucocorticoid therapy. Cyclophosphamide (CYC) was often used as induction therapy in this patient group, but rituximab (RTX) was introduced on ICU from 2008 as a remission-induction agent to treat AAV only. Twenty-seven percent of AAV patients received both CYC and RTX. Plasma exchange (PLEX) and IVIg were available as adjunctive therapies in this patient group. PLEX was frequently used with 71% of the VICU cohort receiving PLEX and 46% of AAV patients receiving PLEX alongside RTX.

### Mortality and prognostic factors

The 30-day and 90-day mortality rates were 15% and 26%, respectively. Comparison of prognostic factors between survivors and non-survivors at 30 and 90 days is presented in Table 2. Analysis combined both AAV and anti-GBM patients, as the small number of anti-GBM patients (*n* = 13) excluded independent analysis; however, these prognostic factors were analysed for AAV patients independently and presented in Supplementary Table S1. Factors associated with 30-day and 90-day mortality in the univariable analysis were: cardiovascular indication for admission (*P* = 0.009 and *P* = 0.033, respectively), gastrointestinal indication for admission (*P* = 0.038 and *P* = 0.011, respectively) and APACHE II score at admission (*P* = 0.020 and *P* = 0.015, respectively). Additionally, admission prior to 2008 was significantly associated with 90-day mortality (*P* < 0.005).

**Table 2:** Univariable analysis of factors affecting mortality at 30 and 90 days (*n* = 85).

	30-day survivors (*n* = 72)	30-day non-survivors (*n* = 13)	*P*-value	90-day survivors (*n* = 63)	90-day non-survivors (*n* = 22)	*P*-value
Patient characteristics						
Age, median (IQR), years	62 (49–71)	65 (29–80)	0.53	61 (45–70)	67 (52–76)	0.17
Male gender, *n* (%)	49 (68)	8 (62)	0.89	42 (67)	15 (68)	1.00
Co-morbid with hypertension, *n* (%)	26 (36)	5 (38)	0.48	20 (32)	11 (50)	0.059
AAV diagnosis, *n* (%)	58 (81)	12 (92)	0.21	50 (79)	20 (91)	0.18
New onset, *n* (%)	60 (83)	8 (62)	0.056	54 (86)	14 (64)	0.081
ICU admission and management						
Length of stay, median (IQR), days	9 (4–22)	8 (1–10)	0.072	8 (3–17)	9 (2–29)	0.65
APACHE II score, median (IQR)	18 (15–23)	24 (19–28)	**0.020**	18 (14–23)	21 (19–25)	**0.015**
Renal indication, *n* (%)	47 (65)	8 (62)	1.00	40 (63)	15 (68)	0.89
Respiratory indication, *n* (%)	62 (86)	11 (85)	1.00	54 (86)	19 (86)	1.00
Cardiovascular indications, *n* (%)	12 (17)	7 (54)	**0.009**	10 (16)	9 (41)	**0.033**
Gastrointestinal indications, *n* (%)	5 (7)	4 (31)	**0.038**	3 (5)	6 (27)	**0.011**
Neurological indications, *n* (%)	7 (10)	1 (8)	1.00	7 (11)	1 (5)	0.63
CVVHDF in ICU, *n* (%)	45 (63)	11 (85)	0.24	38 (60)	18 (82)	0.14
Ventilation in ICU, *n* (%)	65 (90)	13 (100)	0.53	56 (89)	22 (100)	0.24
Inotropic support, *n* (%)	25 (35)	8 (62)	0.15	20 (32)	13 (59)	0.057
Vasculitis specific treatment, *n* (%)						
CYC	48 (67)	2 (15)	**0.0016**	42 (67)	8 (36)	**0.025**
RTX	35 (49)	2 (15)	0.055	32 (51)	5 (23)	**0.042**
PLEX	55 (76)	5 (38)	**0.015**	50 (79)	10 (45)	**0.006**
IVIg	4 (6)	2 (15)	0.49	2 (3)	4 (18)	0.060
Changes to practice, *n* (%)						
Pre-2008	12 (17)	5 (38)	0.15	7 (11)	10 (45)	**0.002**

The bold text signifies results that are statistically significant (*P* ≤ 0.05). Abbreviations: AAV: ANCA-associated vasculitis; APACHE II: Acute Physiology and Chronic Health Evaluation II; CVVHDF: continuous veno-venous haemodiafiltration; CYC: cyclophosphamide; ICU: intensive care unit; IQR, interquartile range; IVIg: intravenous immunoglobulins; PLEX: plasma exchange; RTX: rituximab.

In univariable analyses, 30-day survival was associated with use of cyclophosphamide (*P* < 0.005) as a remission-induction therapy and the use of plasma exchange as an adjunct to treatment (*P* = 0.015). Ninety-day survival was associated with the use of cyclophosphamide (*P* = 0.025), rituximab (*P* = 0.042) and plasma exchange (*P* = 0.006). In univariable analysis of AAV patients only, all trends held.

These associations were not found to be significant when included in multivariable models for 30-day or 90-day mortality; however, there was a trend towards statistical significance (*P* < 0.1) for admission prior to 2008 and receipt of renal support in intensive therapy unit (ITU) as being associated with 90-day mortality (*P* = 0.081 and *P* = 0.051 respectively) (Supplementary Tables S2 and S3). All five patients who received ECMO were alive at the point of end of follow-up.

Cumulative survival to end of follow-up with respect to the use of cyclophosphamide, rituximab and plasma exchange is illustrated in the unadjusted Kaplan–Meier Survival Curves (Figs 1A, B and C, respectively). These demonstrate univariable associations between cyclophosphamide, rituximab and plasma exchange and long-term survival (*P* = 0.044, *P* = 0.009 and *P* < 0.001, respectively).

**Figure 1:**
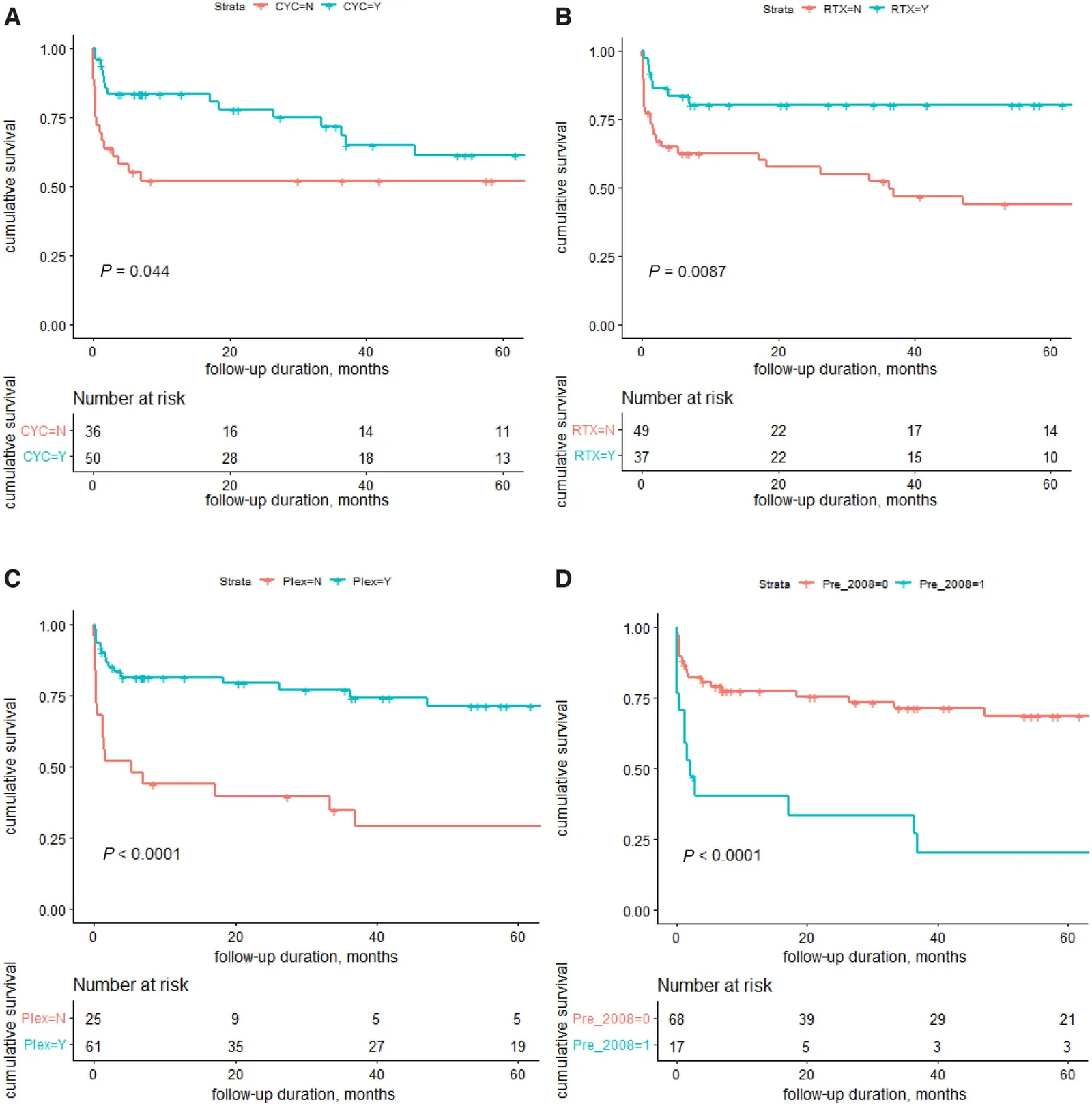
Kaplan–Meier survival curves for long-term survival by treatment received. (**A**) Cyclophosphamide, (**B**) rituximab, (**C**) plasma exchange, (**D**) VICU cohort pre-2008 and post-2008

A multivariable Cox-proportional regression analysis of long-term mortality (co-variates shown in Table 3) found the use of plasma exchange (hazard ratio [HR] 0.38 [95% CI: 0.18, 0.80], *P* = 0.01) were associated with long-term survival.

**Table 3:** Multivariable Cox proportional hazards regression analysis of mortality.

	Mortality: AAV and Anti-GBM (*n* = 86, events = 33)		Mortality: AAV only (*n* = 70, events = 29)	
Covariate	HR (95% CI)	*P*-value	HR (95% CI)	*P*-value
Pre-2008	2.17 (0.77, 6.10)	1.4 × 10^−1^	7.52 (2.74, 20.60)	8.9 × 10^−5^
Age ≥65	1.60 (0.49, 5.30)	4.4 × 10^−1^	5.26 (1.82, 15.20)	2.2 × 10^−3^
Cyclophosphamide	0.48 (0.20, 1.12)	9.0 × 10^−2^	0.62 (0.27, 1.39)	2.5 × 10^−1^
Plasma exchange	0.38 (0.18, 0.80)	1.1 × 10^−2^	0.35 (0.16, 0.74)	6.5 × 10^−3^

Abbreviations: AAV: ANCA-associated vasculitis; CYC: cyclophosphamide; GBM: glomerular basement membrane disease; HR, hazard ratio; PLEX: plasma exchange; RTX: rituximab.

A Cox-proportional regression multivariable analysis was used to assess the association between rituximab use and long-term survival in AAV VICU patients (Table 3), as rituximab is not used anti-GBM disease. Plasma exchange (HR 0.35 [95% CI: 0.16, 0.74], *P* < 0.05) continued to be independently associated with long-term survival in this sub-group, and both age (65 years or greater) and treatment prior to 2008 were associated with long-term mortality (HR 5.26 [95% CI: 1.82, 15.20], *P* < 0.005 and HR 7.52 [95% CI: 2.74, 20.60], *P* < 0.005, respectively).

### New ESKD and prognostic factors

Of the 63 VICU patients who survived 90 days, three had pre-existing ESKD (excluded from further analysis), 17 developed new ESKD and 43 remained RRT independent. Prognostic factors and their relationship to the development of ESKD were explored in Table 4.

**Table 4:** Univariable analysis of factors affecting RRT dependency among 90-day survivors, *N* = 63^a^ (range or percentage).

	RRT independent (*n* = 43)	New RRT dependence (*n* = 17)	*P*-value
Patient characteristics			
Age, median (IQR), years	63 (45–70)	61 (44–72)	0.88
Male gender, *n* (%)	30 (70)	11 (65)	0.94
Diagnosis of AAV, *n* (%)	40 (93)	9 (53)	**<0.005**
New onset, *n* (%)	36 (84)	17 (100)	0.21
ICU admission and management			
Length of stay, median (IQR)	8 (3–18)	8 (3–14)	0.68
APACHE II, median (IQR)	16 (12–20)	23 (19–27)	**<0.005**
Renal indications, *n* (%)	21 (49)	16 (94)	**<0.005**
Creatinine on ICU, µmol/L, median (IQR)	165 (74–349)	668 (485–1007)	**<0.005**
Cardiovascular indications, *n* (%)	8 (19)	2 (12)	0.80
Nephritis, *n* (%)	25 (58)	17 (100)	**0.011**
CVVHDF in ICU, *n* (%)	18 (42)	17 (100)	**<0.005**
Inotropes in ICU, *n* (%)	16 (37)	2 (12)	0.08
Vasculitis specific treatment, *n* (%)			
CYC	27 (63)	12 (71)	0.79
RTX	26 (60)	5 (29)	0.06
PLEX	34 (79)	13 (76)	1.00
IVIg	2 (5)	0 (0)	0.92

a A total of 63 patients survived till 90 days; three patients had pre-existing ESKD dependent on RRT and were excluded from further analysis. The bold text signifies results that are statistically significant (*P* ≤ 0.05). Abbreviations: AAV: ANCA-associated vasculitis; APACHE II: Acute Physiology and Chronic Health Evaluation II; CVVHDF: continuous veno-venous haemodiafiltration; CYC: cyclophosphamide; ESKD: end-stage kidney disease; ICU: intensive care unit; IQR: interquartile range; IVIg: intravenous immunoglobulins; PLEX: plasma exchange; RRT: renal replacement therapy; RTX: rituximab.

Seventy-three percent of anti-GBM patients (8 of 11) developed ESKD and 18% of the AAV (9 of 49) patients developed ESKD. A diagnosis of anti-GBM was associated with the development of ESKD (*P* < 0.005).

Renal failure on admission to ICU (*P* < 0.005), higher creatinine on admission to ICU (*P* < 0.005), higher APACHE II score on admission to ICU (*P* < 0.005), evidence of nephritis (*P* = 0.011) and requirement for renal replacement therapy on ICU (*P* < 0.005) were associated with progression to ESKD. No remission inducing or adjunctive treatments were associated with protection from ESKD. In multivariable analysis, none of these factors on their own were found to lead to or protect against the development of ESKD (Supplementary Table S4).

Development of ESKD was associated with long-term mortality. Seven (41%) of the 17 VICU patients who developed ESKD died before the end of the follow-up period (*P* = 0.01).

### Improving outcomes for vasculitis patients in ICU

The management of vasculitis ICU patients changed in 2008 at Addenbrooke’s Hospital. Seventeen patients were admitted to ICU prior to 2008 and 68 after 2008, likely reflecting an increase in diagnostic rates and regional referrals to the specialist centre. Patients admitted to ICU for the management of vasculitis post-2008 had significantly better 90-day survival than those admitted pre-2008 (*P* = 0.002, Table 2). This observation continued into the long-term survival, where treatment for vasculitis in ICU after 2008 showed favourable outcomes compared with pre-2008 (*P* < 0.001, Fig. 1D).

A case–control analysis was performed to evaluate changes in vasculitis-specific care from any general improvements to ICU care for all patients that would be expected over this time period. VICU patients that were admitted in 2017 or earlier (*n* = 52) were matched with a sample of 192 patients admitted from the same ICU during the same time period, for reasons other than vasculitis treatment. They were matched by age, gender, APACHE II score and year of ICU admission, with a case to control ratio of 1:4. The demographics of both patient groups are reported in Table 5.

**Table 5:** Characteristics of VICU and non-VICU populations (range or percentage)—data inclusive until 2019.

	VICU (*n* = 52)	Non-VICU (*n* = 193)	*P*-value
Admission post-2008, *n*	35	141	0.52
Age, median (IQR), years	54 (17–85)	58 (16–86)	0.14
Male, *n* (%)	32 (62)	102 (53)	0.34
Follow-up, median (IQR), months	28 (0–65)	20 (0–65)	**0.002**
APACHE II scores, median (IQR)	21 (5–31)	22 (7–33)	0.11
Length of ICU stay, median (IQR), days	14 (1–64)	7 (1–76)	**<0.001**
Death in ICU setting, *n* (%)	10 (19)	42 (22)	0.84
Mortality within 30 days, *n* (%)	7 (13)	58 (30)	**0.026**
Mortality within 90 days, *n* (%)	12 (23)	71 (37)	0.064

The bold text signifies results that are statistically significant (*P* ≤ 0.05). Abbreviations: APACHE II: Acute Physiology and Chronic Health Evaluation II; ICU: intensive care unit; IQR: interquartile range; VICU: vasculitis-related intensive care unit.

Univariable analysis of these groups demonstrated that ICU admission for management of vasculitis was associated with a longer length of ICU stay than admission for other conditions (median of 14 days *vs* 7 days, respectively, *P* < 0.001). Admission for management of vasculitis was associated with a lower 30-day mortality (13% *vs* 30%, *P* = 0.026) and 90-day mortality (23% *vs* 37%, *P* = 0.064).

## Discussion

This work sought to characterize the patient population of a large UK cohort of fulminant SVV over 23 years. With respect to the primary outcomes of the study, the 30-day mortality rate was 15% and the 90-day mortality rate was 26%. Twenty-eight percent of 90-day survivors developed new ESKD.

### Mortality

Few studies have reported outcomes of patients admitted to ICU with acute manifestations of SVV. Previously reported mortality rates have varied from 11% to 55% over the last two decades [7–16]. In this study, the 30-day and 90-day mortality rates of 15% and 26%, respectively, were comparable to more recent reports [11–13, 16]. Our study included the sickest patients requiring intensive care only; patients requiring high dependency care level were excluded.

A French cohort of 82 patients receiving both intensive and high dependency level care reported a 90-day mortality of 18% in patients with acute manifestations of SVV [13]. However, in comparison with the French cohort, a greater proportion of the patients in this study required ventilatory support and ECMO, suggesting more severe respiratory involvement. Additionally, the French cohort excluded patients too unwell to receive cyclophosphamide in ICU.

Inclusion of patients with more severe disease in this study will have contributed to higher mortality rates. This is evidenced by the correlation between APACHE II scores and 30 and 90-day mortality. An APACHE II score of 15 or higher is usually associated with multi-organ failure [17]. The median APACHE II score of this cohort was 19, with 75% of patients scoring 15 or above; the majority had multi-organ failure, with over half having concurrent pulmonary and renal failure. Involvement of cardiovascular or gastrointestinal systems were additionally associated with increased risk of 30- and 90-day mortality, in keeping with reported studies [19].

### Treatment to induce remission of SVV

Cyclophosphamide has been shown to improve survival in small-vessel vasculitis and is recommended for life-threatening AAV [2, 19–21]. Additionally, rituximab may be used in combination with low dose cyclophosphamide in fulminant AAV [22] or as an alternative to cyclophosphamide in patients at high infection risk. This study supported these findings, and although underpowered to observe at a multivariable level, univariable associations between both cyclophosphamide and rituximab use and both 30-day and 90-day survival were observed. Furthermore, cyclophosphamide was associated with long-term survival in multivariable Cox proportional hazard analysis for all VICU patients as well as the VICU-AAV subgroup.

Neither cyclophosphamide nor rituximab was used as monotherapy. All patients received glucocorticoids; half of the patients receiving rituximab also received cyclophosphamide; and over two-thirds of patients received plasma exchange in addition to a remission induction therapy—which may affect the generalizability of our findings to other cohorts. This also impacted the ability to identify independent associations from multivariable models for shorter-term survival. Alternative modelling approaches that grouped treatments differently and avoided collinear variables were beyond the scope of the current dataset, but may have produced different results.

We acknowledge the bias in selecting patients for treatment. Patients who died early into their admission may not have had time to receive such treatments and patients too unwell to receive these treatments would have been at increased risk of death. Relationships found between treatment and survival in this study were influenced by survival bias and confounding by indication and cannot establish causality.

### Plasma exchange

In anti-GBM disease, plasma exchange reduces ESKD and mortality and is a standard treatment [5, 21], but its use as an adjunct to treatment in AAV is less clear. The PEXIVAS study [4], a randomized trial of plasma exchange in 704 AAV patients with GFR less than 50 ml/min and/or pulmonary haemorrhage, found that overall plasma exchange did not reduce the rate of mortality or ESKD at 7 years, and was associated with increased infection risk. However, metanalysis of plasma exchange trials found a reduction in risk of ESKD at 12 months in patients presenting with severe renal involvement [23]. In PEXIVAS, 61 patients had severe alveolar haemorrhage, in whom mortality was 8.2% (5/61) with PLEX and 14.8% (9/61) without PLEX [24]. PEXIVAS did not define the role of plasma exchange in ICU AAV patients. Our study is the largest cohort of VICU patients reported to date and found that plasma exchange was associated with long term survival in multivariate analysis for both the whole VICU group and VICU-AAV subgroup. However, relationships found were subject to the same bias as for cyclophosphamide and rituximab and cannot be used to establish causality. International guidelines currently recommend consideration of plasma exchange in renal AAV patients with creatinine >300 μmol/l and/or hypoxic pulmonary haemorrhage [22]. For individual patients the risk of severe infection needs to be considered as well as potential benefit. Adverse outcomes such as sepsis or requiring i.v. antibiotics were noted in this cohort and such outcomes should be collected in future studies to inform relationships to treatment and possible increased risk of mortality.

### Development of ESKD

As with previous studies, we found that renal involvement was associated with mortality. Long-term mortality was associated with acute renal support in ICU as well as the development of ESKD. New cases of ESKD occurred in 28% of ICU survivors, all of whom had had nephritis and required renal support in ICU. Indicators of renal failure (requiring ICU admission for renal failure, requiring renal support in ICU, high creatinine on ICU admission and diagnosis of nephritis) were associated with the development of new ESKD.

Kidney biopsy was not feasible in many critically ill patients due to clinical instability; therefore, renal involvement was clinically adjudicated using the overall vasculitis presentation, laboratory findings and specialist assessment. It is possible that classification of nephritis may have included non-vasculitic causes of acute kidney injury, which is a limitation of this analysis.

Analysis of anti-GBM patients as a sub-group was not possible in this study due to low numbers. Although patients presenting to ICU with renal indications or requiring renal-replacement in ICU did not differ significantly between the total group and AAV subgroup analyses, this study found that anti-GBM patients were more likely to develop new ESKD than AAV patients. This reflects what is seen in literature, where renal outcomes are poor in anti-GBM disease [25–29].

No association was observed between the development of new ESKD and specific therapeutic interventions including rituximab and plasma exchange. This is likely due to a combination of severity of renal involvement in this patient group, small effect size and small sample size.

### Changes in ICU practice

Few other studies have described vasculitis-specific ICU treatments and changes in ICU treatment practice over time [30]. Mortality in this study improved from 2008 on, above and beyond the improvement observed in ICU controls over the same period. Vasculitis is a severe, but potentially reversible, inflammatory condition, where patients may be younger or have fewer baseline comorbidities compared with those admitted to ICU for other causes. The availability of disease-specific treatment has improved over time, with Addenbrooke’s Hospital using rituximab as remission induction therapy for AAV on ICU from 2008 onwards. ICU admission and treatments including CVVHDF and access to ECMO have also evolved over the past two decades.

Benefits of early treatment initiation in both anti-GBM and AAV are well recognized [13]. The Cambridge vasculitis service has expanded, including the introduction of a dedicated vasculitis consultant-led on-call service facilitating early diagnoses and specialist management of inpatients. Increase in vasculitis specialist nurse numbers have facilitated early inpatient administration of infusions and interventions and a 24-h apheresis service has allowed rapid out of hours access to plasma exchange.

### Limitations

This study represents real-world clinical management of patients with life threatening SVV in a tertiary hospital. The limitations of this retrospective study should be emphasized. Only best available medical records dating back 23 years could be used and data were occasionally missing. Incorrect computer based diagnostic coding was also a risk. Every effort was made to limit error through the use of multiple data sources as well as the use of a case-controlled analysis with non-vasculitis matched ICU patients derived from the same databases.

Due to the limitations of the retrospective nature of this study, our observed associations between cyclophosphamide, rituximab and plasma exchange and an improvement in VICU mortality require validation in a prospectively recruited study. The study size limited construction of a predictive model of mortality risk, and confounding by indication and survival bias influenced observed associations, and thus this study cannot be used to derive a protocol for treatment in ICU patients. Further prospective studies in critically ill vasculitis patients are needed to determine whether this patient group may benefit from a more intensive treatment strategy involving combination of cyclophosphamide, rituximab and plasma exchange.

Since collecting data for this study, avacopan has been approved for the treatment of AAV and has been found to an effective steroid-sparing agent that improves kidney function [31, 32]. Including avacopan in treatment strategies for AAV ICU patients is likely to improve outcomes and should be considered in further prospective studies.

## Conclusion

In conclusion, 85 patients with acute manifestations of AAV and anti-GBM disease admitted to ICU with a median age of 63 years and median APACHE II score of 19 had a 26% mortality rate at 90 days, and 28% of survivors developed new ESKD. VICU survival has improved since 2008, associated with the combination of a dedicated on-call vasculitis service, the wider use of rituximab and interventions such as ECMO. This study also highlights the need for further evaluation of treatment regimens in the VICU cohort. The present study provides preliminary data for patient survival and renal recovery, and prognostic factors for future prospective studies.

## Supplementary Material

keag531_Supplementary_Data

## Data Availability

The data underlying this article will be shared on reasonable request to the corresponding author.
