# Supplementary material for: Outcomes of intensive care patients with acute small-vessel vasculitis: a 23-year single-centre experience
Source: Rheumatology (Oxford). 2026 Sep 22;65(10):keag531. doi: 10.1093/rheumatology/keag531 (PMC13671749; doi:10.1093/rheumatology/keag531)
Supplement: Supplementary file 1 [file keag531_supplementary_data.docx]

Supplementary material

**Supplementary Table S1** Univariable analysis of factors affecting mortality at 30 and 90 days for AAV only, N=70 (IQR or percentage)

|  | 30-day survivors, N=58 | 30-day non-survivors, N=12 | *P value* | 90-day survivors, N=50 | 90-day non-survivors, N=20 | *P value* |
| --- | --- | --- | --- | --- | --- | --- |
| **Patient characteristics** | | | | | | |
| Median Age (IQR) | 64 (54-72) | 67 (50-79) | 0.49 | 63 (50-71) | 70 (58-76) | 0.17 |
| Male gender | 43 (74%) | 7 (58%) | 0.45 | 37 (74%) | 13 (65%) | 0.65 |
| Co-morbid with hypertension | 23 (40%) | 5 (42%) | 0.65 | 18 (36%) | 10 (50%) | 0.21 |
| New onset | 48 (83%) | 7 (58%) | 0.074 | 42 (84%) | 13 (65%) | 0.19 |
| **ICU admission and management** | | | | | | |
| Length of stay (IQR) | 10 (4-24) | 8 (2-9) | 0.13 | 8 (4-18) | 9 (4-27) | 0.75 |
| APACHE II score (IQR) | 18 (15-22) | 23 (19-27) | **0.033** | 18 (14-23) | 20 (19-24) | **0.039** |
| Renal indication | 37 (64%) | 8 (67%) | 1.00 | 31 (62%) | 14 (70%) | 0.73 |
| Respiratory indication | 49 (84%) | 10 (83%) | 1.00 | 42 (84%) | 17 (85%) | 1.00 |
| Cardiovascular indications | 11 (19%) | 6 (50%) | 0.056 | 9 (18%) | 8 (40%) | 0.10 |
| Gastrointestinal indications | 5 (9%) | 4 (33%) | 0.064 | 3 (6%) | 6 (30%) | **0.021** |
| Neurological indications | 6 (10%) | 1 (8%) | 1.00 | 6 (12%) | 1 (5%) | 0.66 |
| CVVHDF in ICU | 35 (60%) | 10 (83%) | 0.26 | 28 (56%) | 17 (85%) | 0.054 |
| Ventilation in ICU | 51 (88%) | 12 (100%) | 0.46 | 43 (86%) | 20 (100%) | 0.19 |
| Inotropic Support | 23 (40%) | 8 (67%) | 0.19 | 18 (36%) | 13 (65%) | 0.071 |
| **Vasculitis specific treatment** | | | | | | |
| CYC | 35 (60%) | 2 (17%) | **0.015** | 30 (60%) | 7 (35%) | 0.10 |
| RTX | 35 (60%) | 2 (17%) | **0.015** | 32 (64%) | 5 (25%) | **0.0072** |
| PLEX | 43 (74%) | 4 (33%) | **0.016** | 38 (76%) | 9 (45%) | **0.027** |
| IVIg | 4 (7%) | 2 (17%) | 0.59 | 2 (4%) | 4 (20%) | 0.092 |
| **Changes to Practice** | | | | | | |
| Pre-2008 | 10 (17%) | 4 (33%) | 0.38 | 6 (12%) | 8 (40%) | **0.021** |

*Abbreviations* ICU: intensive care unit; AAV: ANCA-associated vasculitis; APACHE II: Acute Physiology and Chronic Health Evaluation II; CVVHDF: continuous veno-venous haemodiafiltration; CYC: cyclophosphamide; RTX: Rituximab; PLEX: plasma exchange; IVIg: intravenous immunoglobulins. The bold text signifies results that are statistically significant.

**Supplementary Table S2: Multivariable regression analysis of factors affecting mortality at 30 days**

| **Variable** | **Estimate** | **Error** | **P-value** |
| --- | --- | --- | --- |
| (Intercept) | -59.03 | 27852.66 | 0.998 |
| Progressive Disease | 131.05 | 87486.40 | 0.999 |
| Refractory Disease | 64.36 | 82765.78 | 0.999 |
| Relapsed Disease | -20.49 | 27056.52 | 0.999 |
| AAV1 | 41.30 | 17590.78 | 0.998 |
| Nephritis1 | -19.12 | 19350.52 | 0.999 |
| Renal.supportY | 64.92 | 17202.50 | 0.997 |
| APACHE.II.score | -0.25 | 0.25 | 0.320 |
| ITUCVSY | -19.77 | 9930.71 | 0.998 |
| ITUGIY | 42.82 | 13609.68 | 0.997 |
| Pre_20081 | -63.20 | 24951.25 | 0.998 |
| CYCY | -22.04 | 9930.71 | 0.998 |
| RTXY | -22.71 | 9930.71 | 0.998 |
| PlexY | -2.70 | 2.23 | 0.226 |

**Supplementary Table S3: Multivariable regression analysis of factors affecting mortality at 90 days**

| **Variable** | **Estimate** | **Error** | **P-value** |
| --- | --- | --- | --- |
| (Intercept) | -3.47 | 2.30 | 0.131 |
| AAV1 | 1.76 | 1.27 | 0.167 |
| Nephritis1 | -1.63 | 1.28 | 0.204 |
| Renal.supportY | 2.15 | 1.10 | 0.051 |
| APACHE.II.score | 0.06 | 0.08 | 0.405 |
| ITUCVSY | 0.88 | 0.84 | 0.297 |
| Pre_20081 | 1.85 | 1.06 | 0.081 |
| CYCY | -1.08 | 0.77 | 0.162 |
| RTXY | -0.78 | 0.83 | 0.345 |
| PlexY | -0.69 | 0.82 | 0.396 |

**Supplementary Table S4: Multivariable regression analysis of factors affecting ESKD**

| **Variable** | **Estimate** | **Error** | **P-value** |
| --- | --- | --- | --- |
| (Intercept) | -17.18 | 24113.05 | 0.999 |
| Nephritis1 | 36.34 | 10373.10 | 0.997 |
| AAV1 | -39.65 | 20563.27 | 0.998 |
| Renal.supportY | 56.08 | 10961.27 | 0.996 |
| Inotropic.supportY | -19.20 | 6002.51 | 0.997 |
| PlexY | -37.93 | 8316.21 | 0.996 |
| APACHE.II.score | 0.09 | 0.11 | 0.414 |
| creatinine_ICU | 0.00 | 0.00 | 0.999 |
